# The Role of Glutamatergic Neurons in Changes of Synaptic Plasticity Induced by THz Waves

**DOI:** 10.3390/biom15040532

**Published:** 2025-04-04

**Authors:** Lequan Song, Ji Dong, Wenjing Cheng, Zhengjie Fei, Rui Wang, Zhiwei He, Junmiao Pan, Li Zhao, Hui Wang, Ruiyun Peng

**Affiliations:** Beijing Institute of Radiation Medicine, Beijing 100850, China

**Keywords:** terahertz, brain slice, synaptic plasticity, glutamatergic neurons, LTP, GluN2B

## Abstract

**Background**: Terahertz (THz) waves, lying between millimeter waves and infrared light, may interact with biomolecules due to their unique energy characteristics. However, whether THz waves are neurally regulated remains controversial, and the underlying mechanism is elusive. **Methods**: Mouse brain slices were exposed to 1.94 THz waves for 1 h. Synaptic plasticity was evaluated via transmission electron microscopy (TEM), long-term potentiation (LTP), and neuronal class III β-tubulin (Tuj1) and synaptophysin (SYN) expression. Immunofluorescence (IF) and electrophysiology were used to identify neurons sensitive to THz waves. The calcium activity of excitatory neurons, glutamate receptor currents, and glutamate neuron marker expression was also assessed using calcium imaging, a patch clamp, and Western blotting (WB). Optogenetics and chemogenetics were used to determine the role of excitatory neurons in synaptic plasticity impairment after THz wave exposure. NMDA receptor 2B (GluN2B) was overexpressed in the ventral hippocampal CA1 (vCA1) by a lentivirus to clarify the role of GluN2B in THz wave-induced synaptic plasticity impairment. **Results**: Exposure to 1.94 THz waves increased postsynaptic density (PSD) thickness and reduced the field excitatory postsynaptic potential (fEPSP) slope and Tuj1 and SYN expression. THz waves diminished vCA1 glutamatergic neuron activity and excitability, neural electrical activity, and glutamate transporter function. THz waves reduced N-methyl-D-aspartate receptor (NMDAR) current amplitudes and NMDAR subunit expression. Activating vCA1 glutamatergic neurons through optogenetics and chemogenetics mitigated THz wave-induced synaptic plasticity impairment. GluN2B subunit overexpression improved synaptic plasticity marker expression, synaptic ultrastructure, and the fEPSP slope. **Conclusions**: Exposure to 1.94 THz waves decreased synaptic plasticity, glutamatergic neuron excitability, and glutamatergic synaptic transmission in the vCA1. Glutamatergic neuron activation and GluN2B overexpression alleviated THz wave-induced synaptic plasticity impairment; thus, neuromodulation could be a promising therapeutic strategy to mitigate the adverse effects of THz radiation.

## 1. Background

Terahertz (THz) waves are electromagnetic waves with frequencies ranging from 0.1 to 10 THz. Advances in THz technology have led to widespread applications in communications, imaging, biomedicine, security screening, and spectroscopy [1,2]. Despite the promising technological applications of THz waves, their impact on organisms, particularly on the nervous system, is not fully understood. The mechanisms by which THz waves interact with biological tissues include complex biophysical processes, as their energy is concentrated in the vibrational and rotational bands of water molecules, which could cause localized heating effects [3,4,5]. However, studies have suggested that THz waves might also affect cellular function through nonthermal effects, especially in the nervous system [6,7]. Therefore, understanding the potential biological effects of THz waves on the nervous system, particularly on synaptic plasticity, is essential to preventing the potential risks associated with the widespread application of THz technology.

Synaptic plasticity refers to the capacity of synapses to undergo activity-dependent changes in strength, encompassing both the potentiation (strengthening) and depression (weakening) of synaptic responses [8,9]. In the hippocampus, long-term potentiation (LTP) is considered a key neurobiological mechanism of learning and memory. Consequently, the disruption of hippocampal synaptic plasticity could impair cognitive functions, particularly memory [10,11]. As research into the effects of external physical factors, such as electromagnetic waves and radiation, on the nervous system has deepened, synaptic plasticity has become a crucial focus for studying the possible impacts of THz waves. The effects of THz waves on synaptic plasticity remain a subject of debate. Studies have indicated that THz waves might reduce the molecular changes associated with synaptic plasticity in primary hippocampal neurons [12]. In contrast, other studies have suggested that THz wave exposure in rats enhances both the synaptic structure and functional plasticity [13]. Nevertheless, a systematic investigation into the mechanisms by which THz waves affect synaptic plasticity is lacking.

Glutamatergic neurons are the primary excitatory neurons in the brain and are responsible for the majority of excitatory synaptic transmission. The efficiency and plasticity of excitatory synaptic transmission depend largely on glutamate receptors, particularly N-methyl-D-aspartate receptors (NMDARs) and α-amino-3-hydroxy-5-methyl-4-isoxazolepropionic acid receptors (AMPARs). NMDARs are critical for LTP, as well as for synapse formation and stability, whereas AMPARs facilitate fast excitatory synaptic transmission [14]. The disruption of NMDAR and AMPAR functions may directly impact synaptic transmission efficiency, thereby affecting neural network function [15]. Therefore, investigating how THz waves influence glutamatergic neurons and glutamatergic synaptic transmission, especially their effects on NMDARs and AMPARs, will help elucidate the potential neurotoxic mechanisms of THz wave exposure.

Existing studies have indicated that THz waves can affect neuronal bioelectric activity. Some studies have suggested that THz wave exposure alters neuronal membrane potentials, thereby increasing neuronal excitability [16,17]. Additionally, THz waves affect neuronal calcium channels, accelerating the passage of Ca^2+^ through these channels, and the speed of neuronal transmission is positively correlated with the frequency and field strength of THz waves [18]. However, further research is needed to clarify the direct effects of THz waves on specific neuronal types, particularly glutamatergic neurons. In the ventral hippocampal CA1 (vCA1) region, glutamatergic neurons play key roles in learning and memory by regulating excitatory synaptic transmission [19,20]. Thus, studying the effects of THz waves on glutamatergic neurons and their synaptic function may provide crucial insights into how THz wave exposure affects cognitive function.

Although some studies have shown that THz waves could influence neural electrical activity and transmembrane transport, many questions remain regarding their impact on specific neuronal types, synaptic plasticity, and synaptic transmission [21,22]. In particular, whether THz waves affect glutamatergic neuron function via nonthermal mechanisms remains unknown. Given the role of glutamatergic neurons in LTP and synaptic plasticity, they served as an ideal model for studying the effects of THz waves. NMDARs and AMPARs are important glutamate receptors linked to various neurological diseases, and thus investigating the effects of THz waves on these receptors is particularly important [23]. Previous THz time domain spectroscopy studies have discovered characteristic absorption spectra of neurotransmitters. Notably, an absorption peak for glutamate was observed around 1.94 THz, suggesting that there may be specific responses or changes around this frequency [24].

Given the above background, the main purpose of this study is to investigate the effects of 1.94 THz wave exposure on glutamatergic synaptic plasticity and neurotransmission in the vCA1 region. We also used optogenetics and chemogenetics to reveal the roles of glutamatergic neurons in the THz wave-induced impairment of synaptic plasticity. At the molecular level, we focused on the significant changes in the GluN2B subunit and studied how its overexpression affected THz wave-induced alterations in synaptic plasticity. Through this study, we aimed to provide a theoretical basis for assessing the biological safety of THz waves and further elucidate the potential harm caused by THz waves on the nervous system.

## 2. Methods

### 2.1. Animals

All animal studies and experimental procedures were approved by the Animal Care and Use Committee (IACUC-DWZX-2024-574). To avoid the effects of hormone fluctuations in female mice on synaptic plasticity and neuronal activity, C57BL/6N male mice aged 6 weeks were used in this study. The animals were maintained in a conscious and unrestrained state until the start of the experimental procedures. The mice were raised in a specific pathogen-free environment in a laboratory animal facility at temperatures ranging from 21 °C to 23 °C and humidity levels between 40% and 60% on a 12 h light/dark cycle. Standard rodent food and water were freely available to the mice. All experiments were performed exclusively during the daylight period of the light/dark cycle, strictly adhering to institutional guidelines.

### 2.2. Brain Slice Preparation

The mice were deeply anesthetized with pentobarbital sodium and transcardially perfused with 20 mL of oxygenated ice-cold modified artificial cerebrospinal fluid (aCSF) (composition: 10 mM NaCl, 2.5 mM KCl, 1.25 mM NaH_2_PO_4_, 25 mM NaHCO_3_, 0.5 mM CaCl_2_, 7 mM MgCl_2_, 195 mM sucrose, 10 mM glucose, and 2 mM sodium pyruvate; osmolarity 300–305 mOsm/kg, pH 7.35–7.45 adjusted with NaOH). Then, 300 μm-thick coronal sections of vHPC brain were prepared using a vibratome (Campden, UK). For brain slices used for in vitro LTP recording, the hippocampus was first dissected and then sliced. The hippocampus was sliced perpendicular to the long axis to keep the functional connectivity between vCA3 and vCA1 intact. Two adjacent brain slices were obtained from each animal, one of which was assigned to group C and the other to group T. It was ensured that both groups of samples were from the same animal, thus minimizing inter-animal variability. The brain slices were allowed to recover for 30 min in aCSF at 32 °C and then incubated at room temperature for at least 1 h with oxygenated standard aCSF (composition: 119 mM NaCl, 2.5 mM KCl, 1.25 mM NaH_2_PO_4_, 24 mM NaHCO_3_, 2 mM CaCl_2_, 2 mM MgSO_4_, 12.5 mM glucose; osmolarity 300–305 mOsm/kg, pH 7.35–7.45 adjusted with NaOH). All the chemicals used for slice preparation were purchased from Sigma. Detailed information about all reagents and equipment used in this study is provided in Supplementary Table S1.

### 2.3. THz Wave Exposure

A QS1-260-HP source (Microtech Instruments, Eugene, OR, USA) was used to generate THz waves. This quasioptical system uses a QS1-260 back-wave oscillator (BWO) with a frequency range of 114–2349 GHz. In addition, for the BWO-based THz wave source, each frequency corresponds to a specific output power. In this study, we exposed brain slices containing the vCA1 region to THz waves at a frequency of 1.94 THz with a power output of 1 μW for 60 min. During exposure, the slices were continuously perfused with oxygen-saturated aCSF. To prevent the aCSF from absorbing the THz waves, the THz waves were emitted from beneath the chamber. A TPX THz lens (TYDEX, St. Petersburg, Russia) was employed to focus scattered light into a directional beam. The schematic diagram of the THz wave exposure of brain slices is shown in Supplementary Figure S1. Additionally, a fiber-optic temperature monitoring system (Fiso, SPC-HR, Quebec, QC, Canada) was used to track changes in the aCSF temperature during THz wave exposure. A temperature sensor with a sensitivity of 0.01 °C was placed at the bottom of the chamber and submerged in aCSF to ensure precise temperature measurements. The brain slices were divided into two groups: a control (C) group and a THz wave exposure (T) group. The T group was exposed to 1.94 THz radiation for 60 min, whereas the C group was not exposed to THz waves.

### 2.4. Ultrastructural Examination

Samples containing the vCA1 region were collected after THz wave exposure. The brain tissues were fixed in a solution containing 2.5% glutaraldehyde and 4% paraformaldehyde (PFA) at 4 °C for 4 h. Following primary fixation, the samples were washed three times with 0.1 M phosphate buffer and post-fixed in 1% osmium tetroxide for 2 h at room temperature. After post-fixation, the tissues were dehydrated through a graded ethanol series (30%, 50%, 70%, 90%, 100%), cleared in propylene oxide, and embedded in epoxy resin. Ultrathin sections (70 nm thick) were cut from the embedded tissue using an ultramicrotome and stained with uranyl acetate and lead citrate for contrast enhancement. The ultrastructure of the vCA1 region was observed with a transmission electron microscope (TEM, Hitachi, HT7800, Tokyo, Japan). The location of the vCA1 region in the brain atlas in this study is Bregma-2.92 mm, as shown in Supplementary Figure S2. A quantitative analysis of postsynaptic density (PSD) thickness in hippocampal neurons was performed using ImageJ 1.48V software (NIH, Bethesda, MD, USA). Each group was sampled from three mice, and several synaptic structures were randomly selected for observation in each electron microscopic section.

### 2.5. In Vitro LTP Recording

Patch pipettes with a resistance of 1–2 MΩ were filled with aCSF. A brain slice was transferred to the recording chamber where it was positioned and secured with a cover mesh. Oxygenated aCSF at 32 °C was perfused at a rate of 2–4 mL/min for at least 30 min to ensure the stability of the slice before recording. For LTP recordings in the vHPC, a recording electrode was placed in the stratum radiatum of the vCA1 region, while the stimulating electrode was inserted into the vCA3 Schaffer collaterals of the stratum radiatum. The electrode depth, stimulation intensity, and positioning of both electrodes were adjusted to optimize the field excitatory postsynaptic potential (fEPSP) amplitude. Once the maximum fEPSP amplitude was achieved, the stimulation intensity was reduced to 50%, and a 20 min baseline recording was obtained. If the fEPSP amplitude was less than 0.1 mV after repeated adjustments, the brain slice was considered nonviable and discarded. Electrical stimulation was delivered using a stimulator (AM SYSTEM, Model 2100, Sequim, WA, USA), and LTP was induced by applying three 1 s high-frequency stimulation (HFS) trains at 100 Hz, with 20 s intervals between each train. After HFS, fEPSPs were recorded for an additional 30 min to monitor LTP.

### 2.6. Immunofluorescence

Brain slices exposed to THz radiation were fixed in 4% PFA at 4 °C for 12 h. After fixation, the slices were placed in PBS containing 10% goat serum and 0.5% Triton X-100 for 4 h at room temperature. The slices were subsequently incubated overnight at 4 °C with primary antibodies. Detailed information about the antibodies used in immunofluorescence is provided in Supplementary Table S2. The following day, the slices were incubated with fluorescent secondary antibodies (conjugated to Alexa Fluor 488 and Alexa Fluor 546, 1:2000, Invitrogen, Carlsbad, CA, USA) and DAPI at 4 °C overnight. Both primary and secondary antibodies were diluted in a solution of 10% normal goat serum and 0.5% Triton X-100 in PBS. Following the secondary antibody incubation, brain slices were mounted on slides using VECTASHIELD Antifade Mounting Medium (Vectorlabs, H-1700, Newark, CA, USA). Finally, the slices were mounted on slides with coverslips and imaged via a confocal microscope (Oxford Instruments, Dragonfly 200, Oxford, UK). Imaging was performed under consistent parameters, acquiring single optical sections to ensure high resolution for quantitative analysis. The number of cells or the mean optical density was analyzed using ImageJ 1.48V software. The location of the vCA1 region in the brain atlas in this study is Bregma-2.92 mm, as shown in Supplementary Figure S2.

### 2.7. High-Resolution Microelectrode Array (HD-MEA)

The HD-MEA system (Maxwell, MaxOne, Zurich, Switzerland), featuring 26,400 electrodes and 1024 channels integrated within a 4 mm × 2 mm MaxOne chip, was employed to record the synchronous firing activity of vCA1 neurons. To ensure optimal electrode contact, the vCA1 slice was positioned on the HD-MEA and secured with a mesh cover to minimize movement. The slice was continuously perfused with oxygenated aCSF at pH 7.4 and maintained at 33 ± 1 °C. Synchronous neuronal firing activity was induced by applying the GABA_A_ receptor antagonist bicuculline. Neuronal spontaneous activity was assessed using the module of activity scan assay, employing a sparse 7× configuration that consisted of seven configurations for 30 s. The system automatically identified active neurons based on firing frequency and amplitude. Selected active neurons in the target area were recorded for 5 min using the module of network assay. Raw data were processed in MATLAB R2020a to analyze the temporal and spatial distribution of synchronous firing activities, quantify the proportion of spikes within bursts, and calculate the synchronous firing rate.

### 2.8. Western Blot (WB) Analysis

The quantification of protein expression, including sample processing, electrophoretic separation, antibody incubation, washing, detection, and data analysis, was performed using the automated protein analysis system (Bio-Techne, Jess, Minneapolis, MN, USA). This system allows for the efficient and accurate quantification of protein levels. Proteins were extracted from the vCA1 region, and tissue lysis was carried out via a RIPA lysis buffer containing protease inhibitors. Protein concentrations were then determined using the BCA protein quantification method (Thermo, Waltham, MA, USA). Detailed information about the antibodies used in WB is provided in Supplementary Table S2. The secondary antibodies were selected on the basis of species compatibility, and ready-to-use secondary antibody solutions provided by the Jess system were utilized. Following the protocol for the Jess system, samples and reagents were loaded in prefilled plates, and the system automatically performed protein detection. Chemiluminescent signals were captured by the built-in high-sensitivity imaging device. After signal capture, the Jess system generated an electropherogram, and the relative expression of the target proteins was quantified by analyzing the peak areas of the electrophoretic peaks. The final data were processed via the system’s Compass for Simple Western 6.2.0 software, and the results were presented as graphs for further statistical analysis.

### 2.9. Patch Clamp Recording

Electrophysiological recordings of brain slices were performed after the experimental treatments. Patch pipettes with an impedance of 4–6 MΩ were used for patch-clamp recordings and filled with an internal solution containing 130 mM K-gluconate, 5 mM NaCl, 2 mM MgCl_2_, 4 mM Na_2_-ATP, 0.4 mM Na_2_-GTP, 10 mM HEPES, 0.5 mM EGTA, and 10 mM Na_2_-phosphocreatine (osmolarity 300–305 mOsm/kg, pH 7.35–7.45, adjusted with KOH). Before recording, the slices were transferred to a recording chamber and perfused with 32 °C aCSF at 2 mL/min for 20 min for stabilization. Hippocampal neurons were visualized via an upright infrared differential interference contrast (IR-DIC) microscope (Nikon, Tokyo, Japan) equipped with a 40× water immersion objective. Positive pressure was applied to the patch pipettes, which were advanced to the cell surface until a slight depression formed on the membrane. Upon releasing the positive pressure, the resistance increased to over 1 GΩ, and the membrane potential was clamped at –70 mV, establishing a high-resistance seal. In brief, strong negative pressure was applied using a syringe to break the membrane, allowing for whole-cell capacitance and series resistance compensation, resulting in whole-cell recording.

For action potential recordings, the current-clamp mode was used, with stimulation currents ranging from 0 to 200 pA in 10 pA increments over 500 ms. sEPSCs were recorded in voltage-clamp mode after bicuculline (10 μM) was added to the aCSF to block inhibitory synaptic transmission; the potential was clamped at –70 mV, and recordings were made in “gap-free” mode for at least 5 min. For glutamate receptor current recordings, the recording electrode was placed in pyramidal neurons in the vCA1 region, and a stimulating electrode was placed nearby. NMDAR currents were recorded by clamping the membrane potential at +40 mV and adding bicuculline (10 μM) and CNQX (10 μM) to the aCSF. Stimulation was delivered at 0.03 Hz, and the intensity was adjusted to elicit maximal NMDAR currents. An average of 20–30 responses were recorded. AMPAR currents were recorded by clamping the membrane potential at –70 mV and adding bicuculline (10 μM) and D-APV (50 μM) to the aCSF. For extracellular recordings, the electrical signal was recorded while the cell remained sealed.

All electrical signals were acquired using a Multiclamp 700B amplifier (Molecular Devices, San Jose, CA, USA) and digitized at a sampling rate of 20 kHz using a Digidata 1440A (Molecular Devices, San Jose, CA, USA), and data acquisition and analysis were performed using Clampex 10 software (Molecular Devices, San Jose, CA, USA).

### 2.10. Viral Constructs

The following viral vectors were procured from Obio Technology (Obio, Shanghai, China) for this current study: pcSLenti-EGFP-CMV-Grin2b and pcSLenti-EGFP-CMV-MCS. Additionally, rAAV-CaMKIIa-GCaMP8m, rAAV-CaMKIIa-ChrimsonR-mCherry, rAAV-CaMKIIa-hM3Dq-mCherry, and rAAV-CaMKIIa-mCherry were packaged by Brain Case (Brain Case, Shenzhen, China). Detailed information about the viral vectors used in this study is provided in Supplementary Table S3. All viral vectors were stored in aliquots at –80 °C until further use. The virus titers of the AAVs for injection exceeded 10^12^ v.g./mL, and the virus titers of the lentiviruses exceeded 10^8^ TU/mL. Based on the type of virus injected and whether the mice were exposed to THz waves, the mice were divided into the following groups for chemogenetic experiments: the C + mCherry group, T + mCherry group, C + hM3Dq group, and T + hM3Dq group. For the optogenetic experiments, the mice were divided into the C + mCherry group, T + mCherry group, C + ChrimsonR group, and T + ChrimsonR group. Mice overexpressing GluN2B were similarly divided into the C + EGFP group, T + EGFP group, C + OE group, and T + OE group.

### 2.11. Stereotaxic Surgery

The mice were anesthetized by a single intraperitoneal injection (10 mL/kg) of sodium pentobarbital. Each mouse was subsequently secured firmly to a stereotactic frame (Zhongshi Technology, ZS-FDC, Beijing, China). A midline scalp incision was carefully made, and symmetrical craniotomies were created using a microdrill equipped with ultrafine 0.5-mm burrs. Glass micropipettes (with a tip diameter of 10–20 μm) for AAV microinjections were fabricated via a micropipette puller (Lianqi Future, CL-23C, Wuhan, China). These glass micropipettes, initially filled with silicone oil, were connected to a microsyringe pump (WPI, MICRO2T, Sarasota, FL, USA) to achieve complete elimination of air. Virus solutions were injected precisely into the vHPC regions at the following coordinates: −3.15 mm anteroposterior (AP) to bregma, ±3.15 mm lateral to the midline (ML), and −4.25 mm below bregma (DV). AAV solutions were injected into the vHPC at a rate of less than 50 nL/min (0.2 μL/side, bilaterally), and lentivirus solutions were injected into the vHPC at a rate of less than 200 nL/min (2 μL/side, bilaterally). After injection, the glass micropipette was kept in place for 10 min to ensure thorough dispersion of the injected material in the target area. The mice were allowed to recover for at least 3 weeks before subsequent THz wave exposure and experiments.

### 2.12. In Vitro Calcium Imaging

Calcium signals in brain slices were recorded using a CCD camera (Mightex, CXE-C013-U, Pleasanton, CA, USA) connected to an upright fluorescence microscope (Nikon, FN1, Tokyo, Japan). The calcium signals were visualized using the genetically encoded calcium indicator GCaMP8m. The GCaMP8m was excited by light with a wavelength of 470 nm and emitted fluorescence at a wavelength of 510 nm. A brain slice was placed in a perfusion chamber and continuously perfused with oxygenated aCSF at a constant flow rate of 2 mL/min. The chamber was maintained at a temperature of 32 °C throughout the experiment to mimic physiological conditions. Neuronal calcium signal changes were induced by electrical stimulation (100 Hz, 0.1 mA, 0.45 ms, 2 s) [25], and time series images of calcium signals were captured via a high-speed CCD camera. The fluorescence images were analyzed with IAA v0.2.1.40 software (Mightex, Pleasanton, CA, USA), and the changes in the calcium signal are expressed as ΔF/F, where ΔF represents the change in fluorescence intensity and F represents the fluorescence intensity at each time point.

### 2.13. In Vitro Optogenetics

Brain slices containing the vCA1 region were prepared from mice injected with the optogenetic virus (AAV2/9-CaMKIIa-ChrimsonR-mCherry) or the control virus (AAV2/9-CaMKIIa-mCherry). Optogenetic stimulation was performed by an in vitro optogenetic system (Mightex, Polygon1000, Pleasanton, CA, USA) after THz wave exposure to assess whether glutamatergic neuron activation could reverse THz-induced impairment. The stimulation area was selected, and continuous light at 560 nm was directly delivered to the slices. The laser power was set to 10 mW, and each stimulation lasted for 30 s. Simultaneously, cell-attached patch clamp recordings of neurons in the vCA1 region were performed using a multiclamp 700B amplifier. Patch clamp sealing and recording were performed following standard protocols, as described previously. The frequency of extracellular neuronal firing was quantitatively analyzed to assess the effects of optogenetic activation.

### 2.14. In Vitro Chemogenetics

For the chemogenetic experiments, we prepared brain slices containing the vCA1 region from mice injected with the following viruses: rAAV-CaMKIIa-hM3Dq-mCherry or rAAV-CaMKIIa-mCherry (Brain Case, Shenzhen, China). After THz wave exposure, the slices were placed in a perfusion chamber and continuously perfused with oxygenated aCSF containing 5 μM clozapine-N-oxide (CNO, MCE) for 1 h to activate glutamatergic neurons. After chemogenetic activation, LTP, immunofluorescence, and WB analyses were conducted as described previously.

### 2.15. Statistical Analysis

The results were statistically analyzed using SPSS 19.0 software and an independent sample *t* test with 95% confidence intervals. *p* < 0.05 and *p* < 0.01, denoted by * and **, respectively, were considered to indicate statistically significant differences. Sample information for all statistical graphs in this study can be found in Supplementary Table S4.

## 3. Results

### 3.1. THz Waves Impair Synaptic Plasticity in vCA1 Neurons

THz waves have been demonstrated to significantly impair the structure and function of neurons in the vCA1 region, which plays a crucial role in memory consolidation and spatial navigation [26]. Water molecules in tissue strongly absorb THz radiation, potentially leading to thermal effects [27]. However, in our experiments, the temperature increase observed in aCSF was less than 1 °C (ΔT < 1 °C, Supplementary Figure S3), indicating that the impairments caused by THz exposure were not primarily due to thermal effects but rather nonthermal mechanisms.

**Figure 1:**
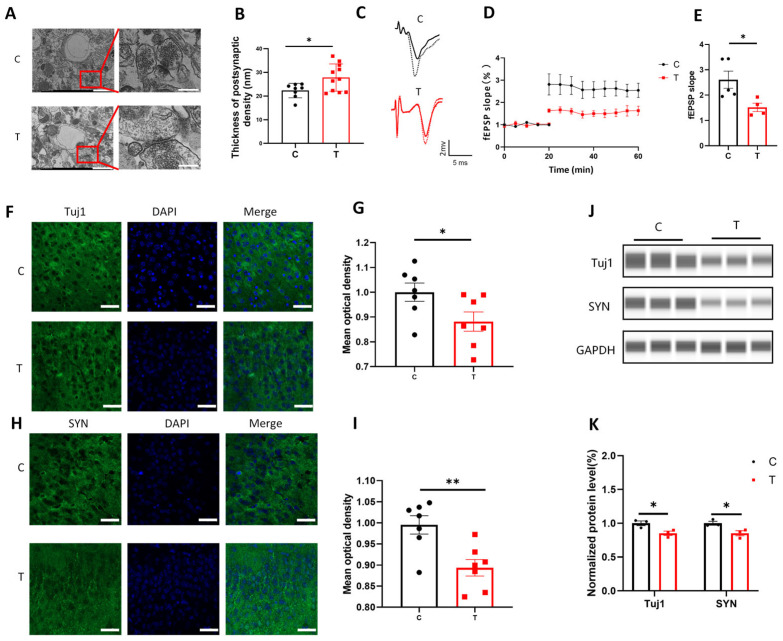
Synaptic plasticity deficits in glutamatergic neurons in the vCA1 region after THz wave exposure. (**A**) Image of vCA1 glutamatergic neurons ultrastructure; scale bar = 1 μm, 250 nm. (**B**) Quantitative analysis of PSD thickness (*n* = 8, 11). (**C**) Representative fEPSP traces in the vHPC after basal stimulation (solid line) and HFS (dashed line); scale bar = 1 mV, 5 ms. (**D**) Changes in the fEPSP slope after HFS. (**E**) Average fEPSP slope relative to basal stimulation for 50–60 min (*n* = 5, 4). (**F**) Image of Tuj1 immunofluorescence; scale bar = 40 μm. (**G**) Statistical analysis of Tuj1 immunofluorescence (*n* = 7, 7). (**H**) Image of SYN immunofluorescence; scale bar = 40 μm. (**I**) Statistical analysis of SYN immunofluorescence (*n* = 7, 7). (**J**) WB analysis of the expression of synaptic plasticity-related molecules. (**K**) Quantitative analysis of WB data for synaptic plasticity-related molecules (*n* = 3, 3). * indicates *p* < 0.05, ** indicates *p* < 0.01.

We first examined the structural integrity of synapses in the vCA1 region using TEM (Figure 1A). TEM revealed a marked increase in the PSD thickness of neurons exposed to THz radiation compared with that in the C group (Figure 1B, *p* < 0.05), indicating alterations in the synaptic architecture that were typically associated with impaired synaptic function. PSD thickening could disrupt the proper alignment of postsynaptic receptors, which was critical for effective synaptic transmission [28,29]. To assess the effects of THz waves on synaptic plasticity, we recorded LTP in the vCA3/vCA1 region in the brain slices (Figure 1C,D). Quantitative analysis of the fEPSP slope at 40–50 min revealed that the fEPSP slope of the T group was significantly lower than that of the C group (Figure 1E, *p* < 0.05), indicating that synaptic plasticity was inhibited.

To further investigate the molecular mechanism by which THz waves impair synaptic plasticity, we examined the expression of two important synaptic proteins: Tuj1 and SYN [30,31]. The expression levels of Tuj1 and SYN in the T group were lower than those in the C group (Figure 1F,G, *p* < 0.05; Figure 1I, *p* < 0.01). We also examined the levels of these two molecules via WB analysis (Figure 1J) and found that the expression levels of Tuj1 and SYN were lower in the T group (Figure 1K, *p* < 0.05, *p* < 0.01). Original Western blot image can be found in Figure S6. These results confirmed that 1.94 THz waves could impair synaptic plasticity in vCA1 neurons.

### 3.2. THz Waves Reduce the Excitability of Glutamatergic Neurons and the Expression of Glutamate Transporters in the vCA1 Region

**Figure 2:**
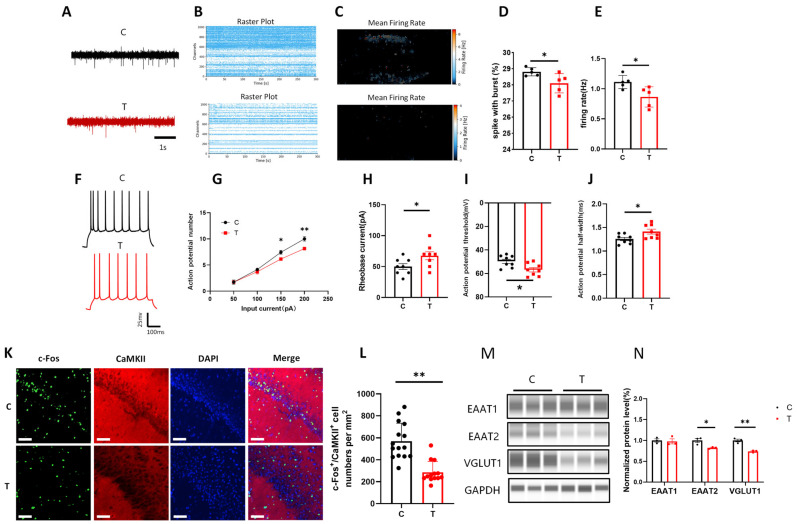
Glutamatergic neuron activity and glutamate transporter expression in the vCA1 region were reduced after THz wave exposure. (**A**) Representative trace of spontaneous extracellular firing in the vCA1 region; scale bar = 1 s. (**B**) Raster plot of firing activity. (**C**) Heatmap of the average firing rate distribution. (**D**) Statistical analysis of the proportion of spikes within bursts (*n* = 5, 5). (**E**) Statistical analysis of the synchronous firing rate (*n* = 5, 5). (**F**) Representative action potential traces of vCA1 neurons; scale bar = 25 mV, 100 ms. (**G**) Statistical analysis of the number of action potentials (*n* = 8, 8). (**H**) Statistical analysis of the rheobase current (*n* = 8, 8). (**I**) Statistical analysis of the action potential threshold (*n* = 8, 8). (**J**) Statistical analysis of the action potential half-width (*n* = 8, 8). (**K**) Immunofluorescence costaining of c-Fos and CaMKII; scale bar = 60 μm. (**L**) Quantitative analysis of the number of immunofluorescent cells (*n* = 15, 13). (**M**) WB analysis of the expression of glutamate transporters. (**N**) Statistical analysis of the WB data for glutamate transporters (*n* = 3, 3). * indicates *p* < 0.05, ** indicates *p* < 0.01.

To further investigate the effects of THz radiation on neuronal activity, we used a high-density microelectrode array to record extracellular firing activity across multiple neurons in the vCA1 region (Figure 2A). The raster plots and heatmaps generated from these recordings revealed that spontaneous neuronal firing was significantly reduced in the T group (Figure 2B,C). Additionally, burst analysis revealed that the proportion of spikes occurring within bursts and the frequency of synchronized firing were both markedly reduced in the T group (Figure 2D,E, *p* < 0.05). This reduction in synchronized firing activity suggested that THz radiation not only affected individual neuronal excitability but also disrupted the coordination of neuronal networks, which was crucial for proper hippocampal function.

We also examined the excitability of vCA1 glutamatergic neurons via patch clamp recording and detected the response to 0–200 pA step current injection in the cells (Figure 2F). Compared with that in the C group, the number of action potentials of neurons in the T group was significantly lower at 150 pA and 200 pA (Figure 2G, *p* < 0.05, *p* < 0.01), whereas the rheobase current (the minimum current required to excite action potentials), the absolute value of the action potential threshold, and the half-width of the rheobase current were significantly greater (Figure 2H–J, *p* < 0.05). This result indicated that after exposure to THz waves, the neurons required stronger stimulation to reach the threshold necessary for triggering action potentials, reflecting diminished neuronal excitability.

Given that 80–90% of cells in the vCA1 region are glutamatergic neurons, we first evaluated changes in glutamatergic neuronal activity [32]. To investigate the effects of THz waves on the activity and excitability of glutamatergic neurons in the vCA1 region, we performed immunofluorescence co-staining for the immediate early gene c-Fos and the glutamatergic neuron marker CAMKII to assess glutamatergic neuron activity. The number of c-Fos^+^/CaMKII^+^ neurons in the T group was significantly lower than that in the C group (Figure 2K,L, *p* < 0.01), suggesting that THz waves significantly reduced the overall activity of glutamatergic neurons in the vCA1 region, which might have a negative impact on hippocampal function.

Additionally, the results of the WB analysis of the expression of glutamate transporters are shown in Figure 2M. The levels of the glutamate transporters EAAT2 and VGLUT1 were reduced in the T group (Figure 2N, *p* < 0.05, *p* < 0.01), suggesting that THz waves disrupted both glutamate receptor function and the transport of glutamate across synapses. Original Western blot image can be found in Figure S7. These molecular changes likely contributed to the observed impairments in synaptic transmission.

### 3.3. THz Waves Inhibit Glutamatergic Synaptic Transmission

**Figure 3:**
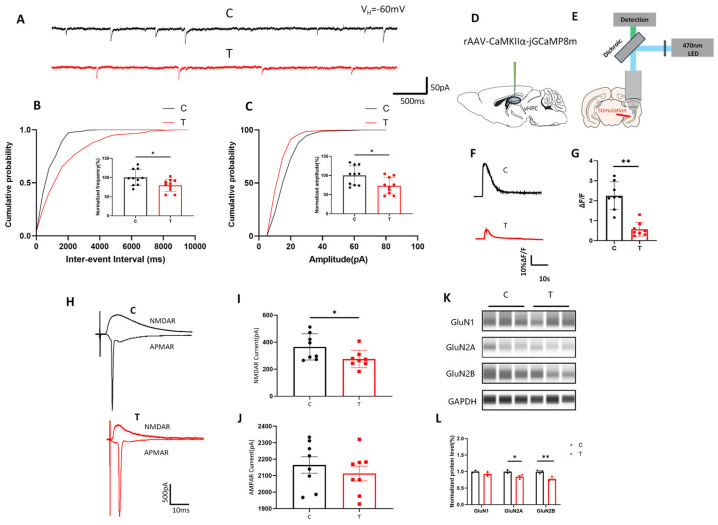
Impaired glutamatergic synaptic transmission in vCA1 neurons after THz wave exposure. (**A**) Representative sEPSC traces in vCA1 neurons. (**B**) Statistical analysis of the sEPSC firing frequency (*n* = 10, 10). (**C**) Statistical analysis of the sEPSC firing amplitude (*n* = 10, 10). (**D**) Schematic diagram of virus injection for calcium imaging. (**E**) Schematic diagram of the neuronal calcium signal recording equipment. (**F**) Representative calcium activity trace in the vCA1 region; scale bar = 10% ΔF/F, 10 s. (**G**) Statistical analysis of the calcium activity amplitude in the vCA1 region (*n* = 8, 8). (**H**) Representative glutamate receptor current traces in vCA1 neurons; scale bar = 500 pA, 10 ms. (**I**) Statistical analysis of NMDAR currents (*n* = 8, 8). (**J**) Statistical analysis of AMPAR currents (*n* = 8, 8). (**K**) WB analysis of the expression of glutamate receptor subunits. (**L**) Statistical analysis of the WB data for glutamate receptor subunits (*n* = 3, 3). * indicates *p* < 0.05, ** indicates *p* < 0.01.

First, we measured spontaneous excitatory postsynaptic currents (sEPSCs) to evaluate the overall synaptic activity of vCA1 neurons (Figure 3A). The frequency and amplitude of sEPSCs in the T group were significantly lower than those in the C group (Figure 3B,C, *p* < 0.05). These findings suggest that THz waves reduced excitatory input to neurons by impairing synaptic transmission.

Given the crucial role of calcium signaling in neuronal excitability and synaptic plasticity [33], we investigated whether THz waves affected calcium transients in glutamatergic neurons in the vCA1 region. We injected a virus for calcium imaging (rAAV-CaMKIIα-GCamp8m) into the vCA1 region and conducted real-time monitoring of calcium transients in glutamatergic neurons three weeks later (Figure 3D). Electrodes were used to stimulate the vCA1 region in brain slices to evoke calcium transients [25], and calcium activity was recorded at an excitation wavelength of 470 nm (Figure 3E). The amplitude of calcium transients in the T group was significantly lower than that in the C group (Figure 3F,G, *p* < 0.01). The decrease in calcium activity indicated that THz radiation disturbed the normal regulation of intracellular calcium concentrations, which played a vital role in synaptic transmission and plasticity. Consequently, the observed decrease in calcium activity might be a key mechanism underlying the THz radiation-induced impairment of synaptic plasticity.

Given the observed reduction in the activity and excitability of glutamatergic neurons in the vCA1 region, we hypothesized that alterations in glutamate receptors might occur. To further investigate the effects of THz waves on glutamatergic synaptic transmission, we measured glutamate receptor currents in the vCA1 region (Figure 3H). At a holding potential of +40 mV, we recorded NMDAR currents by blocking AMPARs with CNQX. Compared with those in the C group, the NMDAR currents in the T group were significantly lower (Figure 3I, *p* < 0.05). However, when we blocked NMDARs with APV at a holding potential of -70 mV to record AMPAR currents, there were no significant differences between the C and T groups (Figure 3J, *p* > 0.05). These results suggested that THz waves could specifically impair NMDAR currents without affecting AMPAR currents.

We also conducted a WB analysis to assess the expression levels of glutamate receptor subunits (Figure 3K). Original Western blot image can be found in Figure S8. The results revealed that the levels of GluN2A and GluN2B in the T group were reduced (Figure 3L, *p* < 0.05, *p* < 0.01), with the decrease in GluN2B expression being more significant. Therefore, we proposed that THz waves impaired glutamatergic synaptic transmission by reducing the expression levels of glutamate receptor subunits.

### 3.4. Activation of Glutamatergic Neurons Reverses THz-Induced Synaptic Impairments

**Figure 4:**
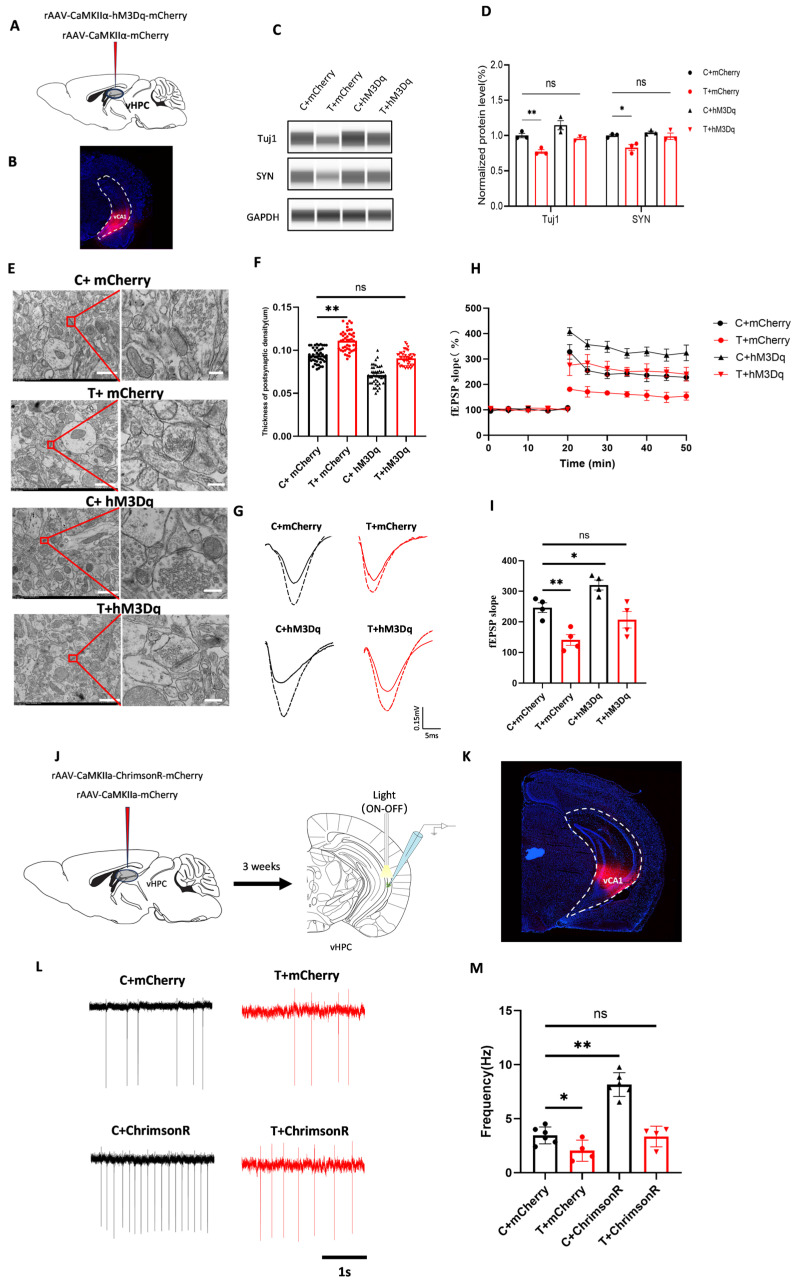
Activation of vCA1 glutamatergic neurons rescues THz-induced synaptic plasticity deficits in vCA1 neurons. (**A**) Schematic diagram of chemogenetic virus injection. (**B**) Verification of the chemogenetic virus injection site. (**C**) WB analysis of the expression of synaptic plasticity-related molecules after chemogenetic activation. (**D**) Quantitative analysis of the WB data for synaptic plasticity-related molecules (*n* = 3, 3, 3, 3). (**E**) Image of vCA1 neurons’ ultrastructure after chemogenetic activation; scale bar = 1 μm, 250 nm. (**F**) Statistical analysis of PSD thickness (*n* = 54, 43, 42, 46). (**G**) LTP recording of vCA1 neurons after chemogenetic activation; scale bar = 0; 15 mV, 5 ms. (**H**) Changes in the fEPSP slope after HFS. (**I**) Average fEPSP slope in response to basal stimulation for 50–60 min (*n* = 4, 4, 4, 4). (**J**) Schematic diagram of the optogenetic experiment. (**K**) Verification of the optogenetic virus injection site. (**L**) Extracellular spontaneous discharges of vCA1 neurons following optogenetic activation; scale bar = 1 s. (**M**) Statistical analysis of the extracellular spontaneous discharge frequency (*n* = 4, 4, 4, 4). * indicates *p* < 0.05, ** indicates *p* < 0.01, ns indicates *p* > 0.05.

Given that THz waves impaired the synaptic plasticity and neuronal activity of glutamatergic neurons, we investigated whether activating vCA1 glutamatergic neurons could reverse the impairment of synaptic plasticity. First, we injected a chemogenetic virus (rAAV-CaMKIIα-hM3Dq-EGFP) or a control virus (rAAV-CaMKIIα-EGFP) into the vCA1 region, leading to the expression of hM3Dq receptors in vCA1 glutamatergic neurons (Figure 4A). The virus injection site was confirmed by the presence of red fluorescence in the vCA1 region (Figure 4B). WB analysis was used to assess the impact of glutamatergic neuron activation on the expression of synaptic plasticity-related molecules. Protein levels were normalized to GAPDH as the loading control. We detected a decreased expression of Tuj1 (Figure 4D, *p* < 0.01) and SYN (Figure 4D, *p* < 0.05) in the T + mCherry group compared with that in the C + mCherry group. Similarly, Tuj1 expression in the T + hM3Dq group was lower than in the C + hM3Dq group (Figure 4D, *p* < 0.01). However, following glutamatergic neuron activation, Tuj1 and SYN expression in the T + hM3Dq group returned to normal levels, showing no significant difference from that in the C + mCherry group (Figure 4D, *p* > 0.05), which indicated that the activation of glutamatergic neurons could reverse the change in the levels of synaptic plasticity-related molecules by THz wave exposure. Original Western blot image can be found in Figure S9. In addition, through immunofluorescence, we evaluated the expression levels of the synaptic plasticity-related molecules Tuj1 and SYN after the glutamatergic neurons were activated (Supplementary Figure S4). Tuj1 and SYN expression levels increased significantly in the C+ hM3Dq group (Supplementary Figure S4B–D, *p* < 0.01) and decreased in the T + mCherry group (Supplementary Figure S4B–D, *p* < 0.01) when compared with those in the C + mCherry group. Activating vCA1 glutamatergic neurons induced the expression levels of these proteins in the T + hM3Dq group to return to a normal state (Supplementary Figure S4B–D, *p* > 0.05). These findings suggest that activating vCA1 glutamatergic neurons could reverse the decrease in synaptic plasticity-related molecule expression after exposure to THz waves.

To investigate the effect of glutamatergic neuron activation on synaptic structural plasticity, we analyzed the neuronal ultrastructure via transmission electron microscopy (Figure 4D, *p* < 0.01). Compared with that in the C + mCherry group, the synaptic density in the T + mCherry group was significantly greater (Figure 4D, *p* < 0.01). However, after the glutamatergic neurons were activated, the synaptic density in the T + hM3Dq group returned to normal, and the synaptic density in the T + hM3Dq group was not significantly different from that in the C + mCherry group (Figure 4D, *p* < 0.01). Additionally, we assessed synaptic functional plasticity by measuring the LTP in brain slices following glutamatergic neuron activation. The fEPSP slope in the T + mCherry group was lower than that in the C + mCherry group (Figure 4D, *p* < 0.01), whereas it was greater in the C + hM3Dq group (Figure 4D, *p* < 0.01). Following glutamatergic neuron activation, the fEPSP slope in the T + hM3Dq group returned to normal, with no significant difference from that in the C + mCherry group (Figure 4D, *p* > 0.05). These results suggest that activating vCA1 glutamatergic neurons could reverse the impairment of synaptic structural and functional plasticity caused by THz waves.

We next used optogenetics combined with patch clamp recording to investigate the effects of activating vCA1 glutamatergic neurons on extracellular firing after THz wave exposure. We injected the optogenetic virus (rAAV-CaMKIIa-ChrimsonR-mCherry) or its control virus (rAAV-CaMKIIa-mCherry) into the vCA1 region, and optogenetic stimulation and patch clamp recording were performed after the virus was stably expressed (Figure 4J,K). After THz wave exposure, we recorded the extracellular firing of vCA1 neurons and analyzed the firing frequency (Figure 4L). The results revealed that the extracellular firing frequency in the T + mCherry group was lower than in the C + mCherry group (Figure 4M, *p* < 0.05), whereas it was greater in the C + hM3Dq group (Figure 4M, *p* < 0.01). After glutamatergic neuron activation, the extracellular firing frequency in the T + hM3Dq group returned to normal, with no significant differences from that in the C + mCherry group (Figure 4M, *p* > 0.05). These findings indicated that THz waves impaired the synaptic plasticity and extracellular firing of vCA1 glutamatergic neurons, whereas the activation of these neurons could reverse the damage caused by THz wave exposure.

### 3.5. GluN2B Overexpression Reverses THz Wave-Induced Synaptic Impairments

**Figure 5:**
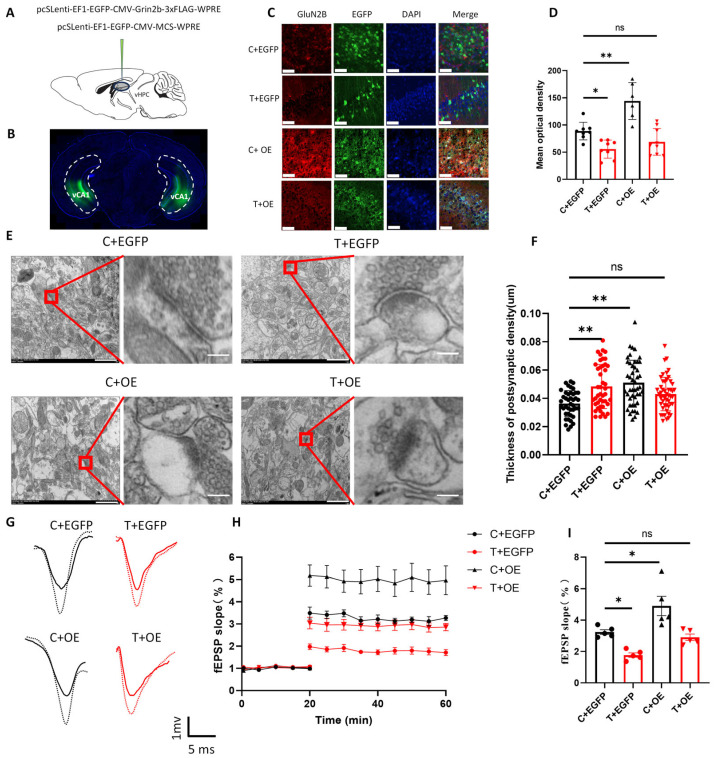
Overexpression of GluN2B in vCA1 glutamatergic neurons can reverse the THz-induced impairment of synaptic plasticity. (**A**) Schematic diagram of GluN2B overexpression via virus injection. (**B**) Verification of the GluN2B overexpression virus injection site. (**C**) Immunofluorescence image showing the effect of GluN2B overexpression; scale bar = 60 μm. (**D**) Statistical analysis of GluN2B immunofluorescence (*n* = 8, 8, 6, 9). (**E**) TEM images of vCA1 neurons after GluN2B overexpression; scale bar = 1 μm, 250 nm. (**F**) Statistical analysis of PSD thickness (*n* = 43, 44, 46, 48). (**G**) LTP of vCA1 neurons after GluN2B overexpression; scale bar = 1 mV, 5 ms. (**H**) Changes in the fEPSP slope after HFS (*n* = 5, 5, 5, 5). (**I**) Average fEPSP slope in response to basal stimulation for 50–60 min (*n* = 5, 5, 5, 5). * indicates *p* < 0.05, ** indicates *p* < 0.01, ns indicates *p* > 0.05.

By measuring the expression of NMDAR subunits, we found that THz waves could reduce the expression of glutamate receptor subunits, especially the GluN2B subunit. To clarify the role of GluN2B in the synaptic plasticity deficits induced by THz wave exposure, we overexpressed the GluN2B gene in the vCA1 region using a lentivirus. We injected a GluN2B-overexpressing virus (pcSLenti-EF1-EGFP-CMV-Grin2b-3xFLAG-WPRE) or its control virus (pcSLenti-EF1-EGFP-CMV-MCS-WPRE) into the vCA1 region (Figure 5A). After 3 weeks, we found that the virus was expressed in the vCA1 region (Figure 5B) and that GluN2B in the C + OE group was overexpressed compared with that in the C + EGFP group (Figure 5C,D, *p* < 0.01), whereas the T + EGFP group presented reduced GluN2B expression (Figure 5D, *p* < 0.05). Following GluN2B overexpression, the GluN2B level in the T + OE group returned to that observed in the C + EGFP group (Figure 5D, *p* > 0.05). Additionally, we assessed the expression levels of the synaptic plasticity-related proteins Tuj1 and SYN via immunofluorescence after GluN2B overexpression (Supplementary Figure S5). Tuj1 and SYN expression levels were greater in the C + OE group (Supplementary Figure S5B,D, *p* < 0.05, *p* < 0.01) and lower in the T + EGFP group (Supplementary Figure S5B,D, *p* < 0.05) than in the C + EGFP group. The overexpression of GluN2B induced the expression levels of these proteins in the T + OE group to return to a normal state (Supplementary Figure S5B,D, *p* > 0.05). These findings suggested that the overexpression of the GluN2B subunit in vCA1 glutamatergic neurons could reverse the reduction in the expression of synaptic plasticity-related molecules caused by THz wave exposure.

Subsequently, we investigated the effects of GluN2B overexpression on synaptic structure and functional plasticity in the vCA1 region using TEM (Figure 5E) and LTP (Figure 5G). The synaptic density significantly increased in the T + EGFP and C + OE groups compared with that in the C + EGFP group (Figure 5F, *p* < 0.01). After GluN2B overexpression, the synaptic density returned to normal levels, showing no significant differences with that in the C + EGFP group (Figure 5F, *p* > 0.05). To assess changes in synaptic plasticity, we measured LTP in brain slices following GluN2B overexpression. We recorded the fEPSP slope after basal stimulation and HFS and performed a quantitative analysis of the fEPSP slope at 40–50 min (Figure 5H). The fEPSP slope in the T + EGFP group was lower than in the C + EGFP group (Figure 5I, *p* < 0.05), whereas it was greater in the C + OE group (Figure 5I, *p* < 0.05). Following GluN2B overexpression, the fEPSP slope returned to normal, with no significant differences from that in the C + EGFP group (Figure 5I, *p* > 0.05). These results suggested that GluN2B overexpression could reverse the deficits in synaptic structure and functional plasticity caused by THz radiation.

## 4. Discussion

Synaptic plasticity refers to dynamic changes in the structural and functional properties of synaptic connections between neurons [34,35]. It serves as the neural foundation for essential brain functions such as adapting to environmental changes, learning, and memory formation. Synaptic plasticity enables the nervous system to adjust its connectivity and transmission efficiency in response to external stimuli and internal needs. In a previous study, rat brain slices were exposed to 0.138 THz radiation, resulting in a continuous increase in the PSP slope within 60 min of exposure, and Golgi staining revealed an increase in the dendritic spine density, indicating enhanced synaptic plasticity at both the structural level and the functional level [13]. PSD95 and SYN are essential synapse-associated proteins [36]. Previous studies have demonstrated that exposure to 0.16 THz waves with an intensity of 50 mW can reduce SYN expression in primary hippocampal neurons. Similarly, exposure to 0.17 THz waves at 10 mW can decrease PSD95 expression in primary cortical neurons. Furthermore, the reduction in the expression of these synapse-related proteins was positively correlated with the exposure duration [37]. Primary hippocampal neurons were exposed to THz waves at a frequency of 2 THz and a power of 3.5 μW for 60 min. This exposure enhanced synaptic transmission. It also upregulated the expression of synaptic plasticity-related molecules, including rSec6, GluN2B, and PSD95 [38]. Other studies have shown that low-power THz waves (0.06 THz, 1 μW/cm^2^) reduce neuronal firing frequency and membrane input resistance. These effects may contribute to decreased neuronal excitability [39]. In our study, we observed that exposure to 1.94 THz radiation impaired synaptic plasticity in vCA1 neurons, as evidenced by increased synaptic density and reduced LTP under electron microscopy. At the molecular level, the expression of the synaptic plasticity-related proteins Tuj1 and SYN was downregulated after THz exposure. The expression of Tuj1, a neuronal marker, and SYN, a synaptic vesicle protein crucial for neurotransmitter release, was significantly affected. The observed increase in PSD thickness after THz wave exposure likely represents a compensatory response to synaptic dysfunction, rather than an enhancement of synaptic plasticity. This hypothesis is supported by the reduction in LTP, which indicates a functional deficit in synaptic plasticity. Furthermore, the downregulation of synaptic proteins such as Tuj1 and SYN suggests that the thickened PSD is insufficient to support effective synaptic transmission. Thus, we concluded that THz waves may impair synaptic plasticity by disrupting the expression of key molecules involved in synaptic structure and function. It is important to note that all observed changes occurred under non-thermal conditions. While the mechanism of tissue impairment resulting from thermal effects is well-established, this study suggests that the non-thermal interaction between THz waves and biomacromolecules may play a more significant role in the observed synaptic plasticity impairment.

To date, research on the effects of THz waves on neurons has focused primarily on nonspecific neuronal populations, with relatively few studies investigating specific neuron subtypes, such as glutamatergic neurons [40]. c-Fos is an immediate early gene that is rapidly expressed following neuronal stimulation and serves as a marker of neuronal activity [41]. The impact of THz waves on neuronal activity is still controversial. Researchers previously reported that the application of 53.6 THz and 9 mW THz waves to thinned mouse skulls for 20 s resulted in an increased proportion of c-Fos^+^ cells in the cortex. Additionally, single-cell loose patch recordings revealed an increase in the firing rate of cortical neurons [42]. In another study, zebrafish were exposed to 2.52 THz waves with an amplitude of 50 mW/cm^2^ for 20 min, and the results showed that neuronal activity was significantly increased and the expression of dopamine-related genes was increased, resulting in elevated neuronal excitability [43]. After exposing primary hippocampal neurons to 0.12 THz waves at 10 mW for 10 min, neuronal activity was assessed with a CCK-8 assay. The results demonstrated a significant reduction in neuronal activity, indicating that THz wave exposure adversely affected cellular function [12]. In our study, THz waves significantly inhibited the activity and excitability of glutamatergic neurons in the vCA1 region. This was demonstrated by reduced c-Fos expression, decreased action potential firing, impaired synchronous firing activity, and reduced calcium signaling. These changes suggest that THz waves might have a negative impact on neuronal excitability and neural network function, potentially affecting brain processes related to learning and memory. This study not only advances the limited research on the effects of THz waves on specific neuronal subtypes but also highlights that the frequency-dependent effects of THz waves on neuronal function may vary across different neuronal types. While previous studies have suggested that low-frequency THz waves generally impair neuronal function and high-frequency THz waves enhance it, our findings on 1.94 THz wave effects in glutamatergic neurons reveal a more complex relationship. This complexity underscores the need for further investigation into the specific characteristics and responses of different neuronal populations to THz waves.

Glutamatergic synaptic transmission is the primary contributor to excitatory transmission in the central nervous system and plays a crucial role in processing and transmitting large amounts of information in the brain [44,45]. Glutamate, the most common excitatory neurotransmitter in the central nervous system, is released by presynaptic neurons and binds to specific receptors on postsynaptic neurons to mediate neural signal transmission [46]. After THz waves with a frequency of 30–45 THz were applied to mouse prefrontal cortex slices, the frequency and amplitude of EPSCs and IPSCs significantly increased, which suggested that THz waves increase excitatory and inhibitory synaptic transmission [47]. Another study reported that exposing primary hippocampal neurons to 3.1 THz waves at 70 μW/cm^2^ for 15 min per day over 3 d increased the frequency of neuronal sEPSCs without significant changes in amplitude [17]. Growth cones are dynamic structures located at the tips of neuronal axons and dendrites that guide neurons to establish precise connections with target cells. These structures detect environmental signals to direct neurite extension, synapse formation, and neural network remodeling, playing crucial roles in synaptic transmission [48]. Research has shown that exposing snail neurons to 3.68 THz waves with an intensity of 15 mW/cm^2^ disrupted growth cone formation and neural network regeneration, indicating the potential inhibition of synaptic transmission and normal neuronal connectivity [49]. In this study, after exposure to THz waves, the NMDAR current amplitude was significantly reduced, whereas AMPAR currents remained unaffected, suggesting that THz waves specifically inhibit NMDAR function without influencing AMPARs. Furthermore, the frequency of sEPSCs decreased, indicating that THz waves interfered with presynaptic glutamate release and weakened signal transmission between neurons. The expression levels of glutamate receptor subunits (GluN1, GluN2A, and GluN2B) and glutamate transporters (EAAT2 and VGLUT1) were significantly reduced, diminishing the efficiency of glutamatergic synaptic transmission. NMDAR subunits, particularly GluN2B, are essential for synaptic plasticity and excitatory signaling, and their downregulation likely impairs synaptic transmission and long-term potentiation. Reduced VGLUT1 expression suggests compromised presynaptic glutamate packaging and release, further weakening synaptic signaling. Similarly, decreased EAAT2 levels, critical for glutamate clearance from the synaptic cleft, may disrupt glutamate homeostasis, potentially increasing excitotoxicity or disturbing the balance of synaptic signaling. These changes are interconnected; reduced presynaptic glutamate release (indicated by lower VGLUT1) diminishes postsynaptic NMDAR activation, weakening synaptic transmission. Additionally, impaired EAAT2 function may exacerbate NMDAR dysfunction and further disrupt synaptic signal transduction. The interplay between presynaptic and postsynaptic alterations likely underlies the observed reduction in synaptic efficiency and plasticity following exposure to 1.94 THz waves. The observed reduction in Tuj1 and SYN further underscores the impact of THz waves on synaptic architecture and vesicle cycling, which are integral to neurotransmitter release and synaptic stability. In contrast to prior studies that predominantly reported the positive effects of THz waves on synaptic transmission under various conditions, our findings reveal a specific inhibitory effect of 1.94 THz waves on glutamatergic synaptic transmission.

THz waves reduced the activity of glutamatergic neurons and impaired the function of glutamatergic receptor subunits. Therefore, we investigated whether activating glutamatergic neurons or overexpressing GluN2B could reverse the impairment of synaptic plasticity induced by THz waves by injecting an AAV or lentiviral vector into the vCA1 [50,51]. We first employed chemogenetic and optogenetic methods to activate glutamatergic neurons. Following chemogenetic activation, changes in the expression of synaptic plasticity-related molecules and the impairment of synaptic plasticity caused by THz waves were reversed. Similarly, light activation reversed the reduction in extracellular firing frequency induced by THz waves. Additionally, the lentiviral overexpression of GluN2B reversed the THz wave-induced changes in the expression of synaptic plasticity-related molecules and the impairment of synaptic plasticity. These results suggest that although THz waves significantly impaired neuronal function, these effects can be effectively reversed by activating glutamatergic neurons or overexpressing GluN2B.

**Figure 6:**
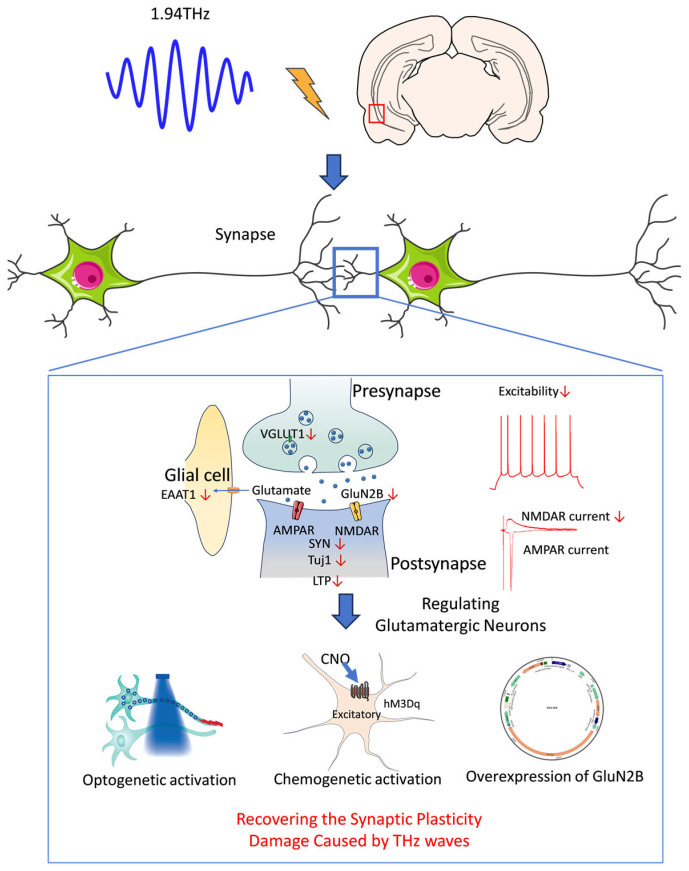
Schematic diagram of the mechanism by which THz waves impair synaptic plasticity by affecting glutamatergic neurons.

The results of this study demonstrated the multiple damaging effects of THz waves on the structure and function of neurons in the vCA1 and revealed their complex effects on synaptic plasticity, neuronal excitability, and glutamatergic synaptic transmission. First, THz wave exposure decreased the expression of the synaptic plasticity-related molecules Tuj1 and SYN, leading to structural and functional damage to synapses. Additionally, THz waves specifically weakened NMDAR currents and downregulated the expression of glutamate receptor subunits and transporters, indicating that the glutamatergic synaptic transmission deficits were closely linked to receptor and transporter dysfunction. THz waves also exerted an overall inhibitory effect on neuronal activity and synaptic network coordination. Genetic manipulation of glutamatergic neurons and overexpression of GluN2B restored synaptic functions, further highlighting the causal relationship between these factors and suggesting potential therapeutic targets (Figure 6). This study underscores the damaging effects of THz waves on hippocampal synaptic plasticity and neuronal function. Understanding these mechanisms can guide the safe application of THz technology and protect humans from the potential effects of radiation exposure on the nervous system.

While this study provides important insights into the effects of THz waves on glutamatergic neurons, which play a key role in maintaining neuronal network balance and regulating excitatory inputs, this study did not address the potential effects of THz waves on GABAergic neurotransmission. In addition, our results suggest a non-thermal mechanism for THz-induced impairment but the specific molecular and cellular pathways remain unidentified. The use of in vitro models, while controlled, may not fully capture the complexity of in vivo interactions, including the contribution of glial cells and broader network dynamics. Further in vivo studies will be essential to validate these findings and evaluate the interplay between excitatory and inhibitory systems under THz wave exposure.

## 5. Conclusions

In conclusion, 1.94 THz waves reduced synaptic plasticity and excitability, impairing synaptic transmission in the vCA1 glutamatergic neurons. Activating glutamatergic neurons and overexpressing the GluN2B receptor subunit could alleviate the THz-induced impairment of synaptic plasticity.

## Data Availability

All data are available in the main text or the Supplementary Materials.

## Supplementary Materials

The following supporting information can be downloaded at: https://www.mdpi.com/article/10.3390/biom15040532/s1, Table S1: Details of reagents and equipment used in the study; Table S2: Details of primary and secondary antibodies used in immunofluorescence and WB; Table S3: Details of viral vectors used in this study; Table S4: Sample information summary in Figures; Figure S1: Schematic diagram of THz waves exposure of mouse brain slice; Figure S2: Anatomical location of the vCA1 region in the brain atlas in this study; Figure S3: Temperature changes over 60 min of exposure to 1.94 THz waves; Figure S4: Activation of vCA1 glutamatergic neurons reverses the THz wave-induced decrease in the expression of synaptic plasticity-related molecules; Figure S5: Overexpression of GluN2B in vCA1 glutamatergic neurons reverses the THz wave-induced decrease in the expression of synaptic plasticity-related molecules; Figure S6: Original Western blot image of Figure 1J; Figure S7: Original Western blot image of Figure 2M; Figure S8: Original Western blot image of Figure 3K; Figure S9: Original Western blot image of Figure 4C.
