# Supplementary material for: The Role of Glutamatergic Neurons in Changes of Synaptic Plasticity Induced by THz Waves
Source: Biomolecules. 2025 Apr 4;15(4):532. doi: 10.3390/biom15040532 (PMC12024624; doi:10.3390/biom15040532)
Supplement: Supplementary file 1 [file biomolecules-15-00532-s001.zip › biomolecules-3489008-supplementary.pdf]

Table S1. Details of reagents and equipment used in the study.

Category	ltem/Equipment	Supplier	Catalog Number/Model	Application
Reagents	RIPA Lysis Buffer	Beyotime Biotechnology	P0013B	Protein extraction
Reagents	Protease inhibitor cocktail	Beyotime Biotechnology	P1005	Preventing protein degradation
Reagents	BCA protein assay kit	Thermo Fisher	23227	Protein quantification
Reagents	Clozapine-N-oxide (CNO)	MCE	HY-17366	Chemogenetic activation
Reagents	Bicuculline	MCE	HY-N0219	GABAA receptor antagonist
Reagents	CNQX	MCE	HY-18708	AMPAR antagonist
Reagents	D-APV	MCE	HY-100735	NMDAR antagonist
Equipment	THz source	Microtech Instruments	QS1-260	Generation of THz waves
Equipment	Vibrating microtome	Campden Instruments	7000smz	Slice preparation
Equipment	TPX THz lens	TYDEX	N/A	Focusing scattered THz waves
Equipment	CCD camera	Mightex	CXE-C013-U	Calcium imaging
Equipment	Polygon optogenetic stimulation system	Mightex	Polygon1000	Light stimulation for optogenetics
Equipment	Upright microscope	Nikon	FN1	Imaging and patch-clamp recordings
Equipment	Confocal microscope	Oxford Instruments	Dragonfly200	Imaging for immunofluorescence
Equipment	Automated protein analysis system	Bio-Techne	Jess	Protein detection and quantification
Equipment	12-230 kDa Separation Module	Bio-Techne	SM-W001	Protein separation for Western blot analysis
Equipment	Transmission electron microscope	Hitachi	HT7800	Ultrastructure observation
Equipment	Fiber-optic temperature monitoring system	Fiso	SPC-HR	Precise temperature trackin
Equipment	HD-MEA system	Maxwell	MaxOne	Recording synchronous firing activity
Equipment	Stereotactic frame	Zhongshi Technology	ZS-FDC	Precise viral vector injection
Equipment	Microsyringe pump	WPI	MICRO2T	Accurate injection of viral vectors
Equipment	Micropipette puller	Lianqi Future	CL-23C	Fabrication of glass micropipettes
Equipment	Multiclamp amplifier	Molecular Devices	700B	Patch-clamp recordings
Equipment	Digidata 1440A	Molecular Devices	1440A	Data digitization
Equipment	Isolated Stimulator	A-M Systems	Model 2100	Electrical stimulation

Table S2. Details of primary and secondary antibodies used in immunofluorescence and WB.

Antibody type	Antibody name	Supplier	Catalog number	Reference	Clonality	Isotype	Immunogen	Concentration	Application
Primary Antibody	c-Fos	Abcam	ab214672	Roy DS et al. (2022).	monoclonal	IgG	Recombinant fragment	1:200	Immunofluorescence
Primary Antibody	Tuj1	Abcam	ab18207	Wahl AM et al. (2023). Sanchez-Bretano A et al. (2023).	polyclonal	IgG	Synthetic peptide	1:1000<br>1:50	Immunofluorescence<br>WB
Primary Antibody	SYN	Abcam	ab32127	Chen P et al. (2023). Xiong J et al. (2023).	monoclonal	IgG	Synthetic peptide	1:2000<br>1:1000	Immunofluorescence<br>WB
Primary Antibody	GluN1	Abcam	ab109182	Tripathi MK et al. (2024).	monoclonal	IgG	Synthetic peptide	1:50	WB
Primary Antibody	GluN2A	Abcam	ab133265	Yokokawa T et al. (2023).	monoclonal	IgG	Recombinant fragment	1:50	WB
Primary Antibody	GluN2B	Abcam	ab183942	Yokokawa T et al. (2023).	monoclonal	IgG	Synthetic peptide	1:50	WB
Primary Antibody	EAAT1	Abcam	ab240235	N/A	monoclonal	IgG	Synthetic peptide	1:50	WB
Primary Antibody	EAAT2	Abcam	ab205248	Liang T et al. (2024).	monoclonal	IgG	Synthetic peptide	1:50	WB
Primary Antibody	VGLUT1	Abcam	ab227805	Du K et al. (2022).	monoclonal	IgG	Synthetic peptide	1:50	WB
Secondary Antibody	Alexa Fluor® 488	Abcam	ab150077	Lin Z et al. (2024).	N/A	IgG	Not available	1:1000	Immunofluorescence
Secondary Antibody	Alexa Fluor® 647	Abcam	ab150083	Meng P et al. (2024).	N/A	IgG	Not available	1:1000	Immunofluorescence
Secondary Antibody	Anti-Rabbit Detection Module	ProteinSimple	DM-001	Wang D et al. (2020)	N/A	IgG	Not available	N/A	WB

Table S3. Details of viral vectors used in this study.

Viral Vector	Supplier	Application	Transgene	Optogenetic/Chemogenetic System
rAAV-CaMKIIa-GCaMP8m	Brain Case	Calcium imaging	GCaMP8m	N/A
rAAV-CaMKIIa-ChrimsonR-mCherry	Brain Case	Optogenetic activation	ChrimsonR	Red light activation
rAAV-CaMKIIa-hM3Dq-mCherry	Brain Case	Chemogenetic activation	hM3Dq	CNO activation
rAAV-CaMKIIa-mCherry	Brain Case	Calcium imaging	mCherry	N/A
pcSLenti-EGFP-CMV-Grin2b	Obio Technology	GluN2B overexpression	Grin2b	N/A
pcSLenti-EGFP-CMV-MCS	Obio Technology	Control vector	EGFP	N/A

Table S4. Sample information summary in Figures.

Figures	Groups	Number of Animals	Number of Brain Slices	Sample Size	Plot Representation
Figure 1B	C	3	3	8	Each point represent a synapse
	T	3	3	11	
Figure 1E	C	5	5	5	Each point represent a slice
	T	4	4	4	
Figure 1G, I	C	7	7	7	Each point represent a slice
	T	7	7	7	
Figure 1K	C	3	3	3	Each point represent a slice
	T	3	3	3	
Figure 2D-E	C	5	5	5	Each point represent a slice
	T	5	5	5	
Figure 2G-I	C	8	8	8	Each point represent a slice
	T	8	8	8	
Figure 2L	C	15	15	15	Each point represent a slice
	T	13	13	13	
Figure 2N	C	3	3	3	Each point represent a slice
	T	3	3	3	
Figure 3B-C	C	10	10	10	Each point represent a slice
	T	10	10	10	
Figure 3G, I, J	C	8	8	8	Each point represent a slice
	T	8	8	8	
Figure 3L	C	3	3	3	Each point represent a slice
	T	3	3	3	
Figure 4D	C+mCherry	3	3	3	Each point represent a slice
	T+mCherry	3	3	3	
	C+hM3Dq	3	3	3	
	T+hM3Dq	3	3	3	
Figure 4F	C+mCherry	3	3	54	Each point represent a synapse
	T+mCherry	3	3	43	
	C+hM3Dq	3	3	42	
	T+hM3Dq	3	3	46	
Figure 4I, M	C+mCherry	4	4	4	Each point represent a slice
	T+mCherry	4	4	4	
	C+hM3Dq	4	4	4	
	T+hM3Dq	4	4	4	
Figure 5D	C+EGFP	8	8	8	Each point represent a slice
	T+ EGFP	8	8	8	
	C+OE	6	6	6	
	T+OE	9	9	9	
Figure 5F	C+EGFP	3	3	42	Each point represent a synapse
	T+ EGFP	3	3	44	
	C+OE	3	3	46	
	T+OE	3	3	48	
Figure 5I	C+EGFP	5	5	5	Each point represent a slice
	T+ EGFP	5	5	5	
	C+OE	5	5	5	
	T+OE	5	5	5	
Supplementary Figure 4 B, D	C+mCherry	5	5	5	Each point represent a slice
	T+mCherry	5	5	5	
	C+hM3Dq	5	5	5	
	T+hM3Dq	5	5	5	
Supplementary Figure 5 B	C+EGFP	7	7	7	Each point represent a slice
	T+ EGFP	8	8	8	
	C+OE	9	9	9	
	T+OE	7	7	7	
Supplementary Figure 5 D	C+EGFP	5	5	5	Each point represent a slice
	T+ EGFP	5	5	5	
	C+OE	9	9	9	
	T+OE	7	7	7	

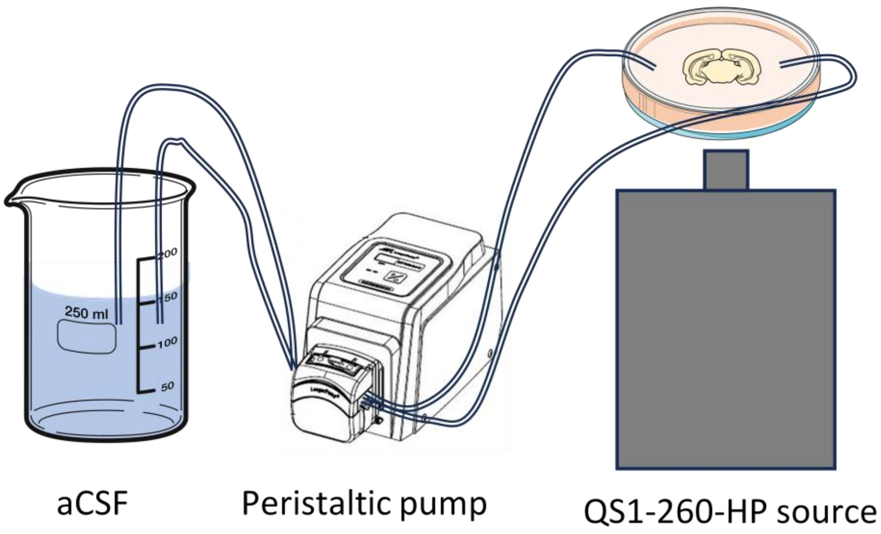

Figure S1. Schematic diagram of THz waves exposure of mouse brain slice.

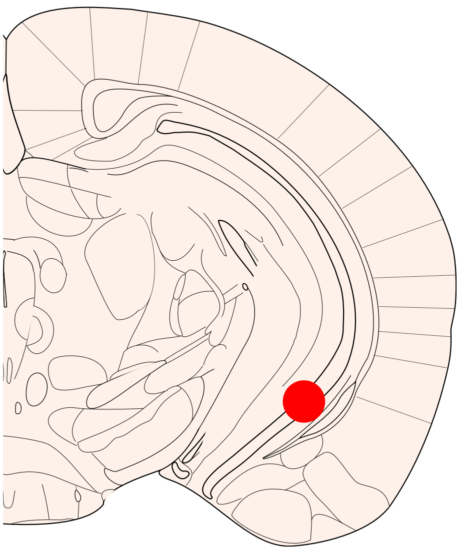

Figure S2. Anatomical location of the vCA1 region in the brain atlas in this study.

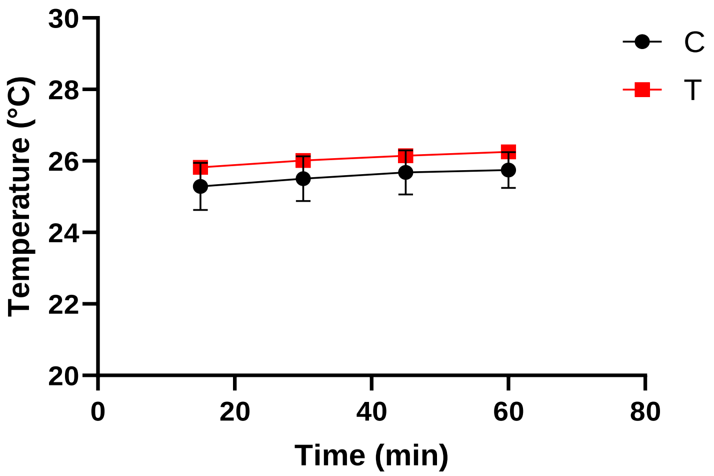

Figure S3. Temperature changes over 60 min of exposure to 1.94 THz waves.

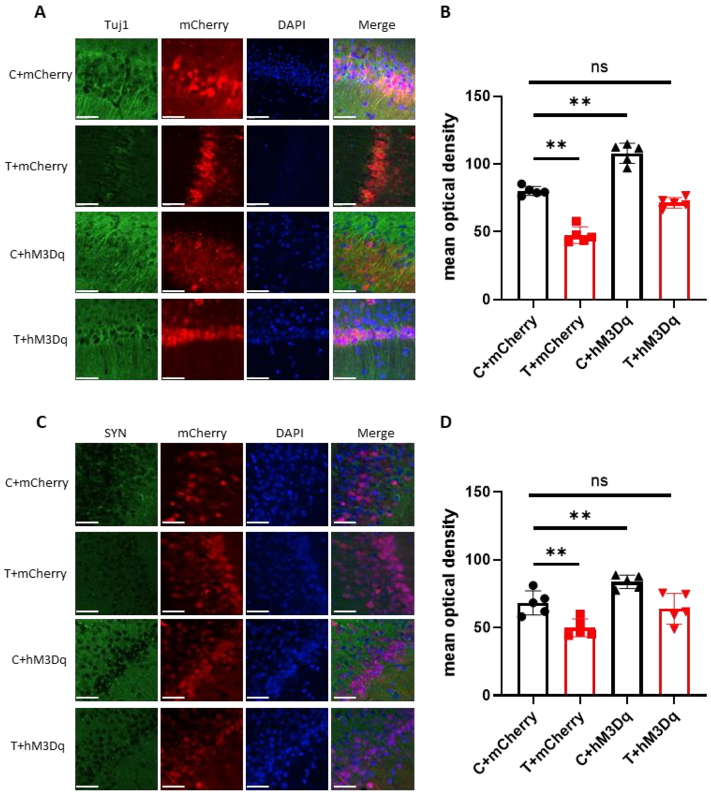

Figure S4. Activation of vCA1 glutamatergic neurons reverses the THz wave-induced decrease in the expression of synaptic plasticity-related molecules. (**A**) Images of Tuj1 immunofluorescence; scale bar = 60 μm. (**B**) Statistical analysis of GluN2A immunofluorescence (n=5, 5, 5, 5). (**C**) Images of SYN immunofluorescence; scale bar = 60 μm. (**D**) Statistical analysis of GluN2B immunofluorescence (n=5, 5, 5, 5). ** indicates P < 0.01, ns indicates P > 0.05.

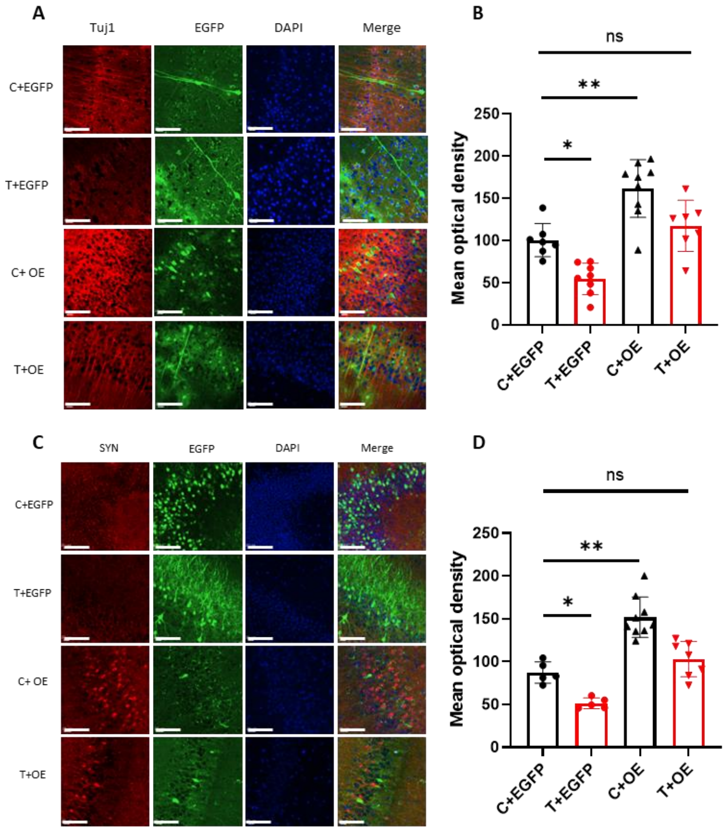

Figure S5. Overexpression of GluN2B in vCA1 glutamatergic neurons reverses the THz wave-induced decrease in the expression of synaptic plasticity-related molecules. (**A**) Images of Tuj1 immunofluorescence; scale bar = 80 μm. (**B**) Statistical analysis of GluN2A immunofluorescence (n=7, 8, 9, 7). (**C**) Images of SYN immunofluorescence; scale bar = 80 μm. (**D**) Statistical analysis of GluN2B immunofluorescence (n=5, 5, 9, 7). * indicates $P < 0.05$, ** indicates $P < 0.01$, ns indicates $P > 0.05$.

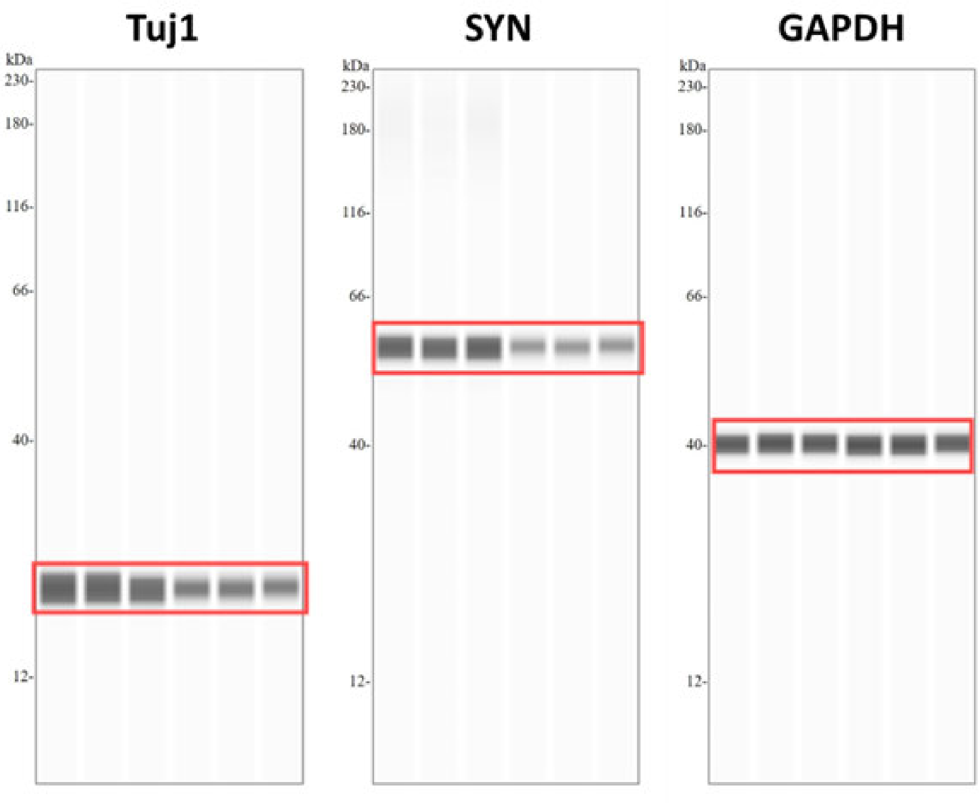

Figure S6. Original Western blot image of Figure 1J.

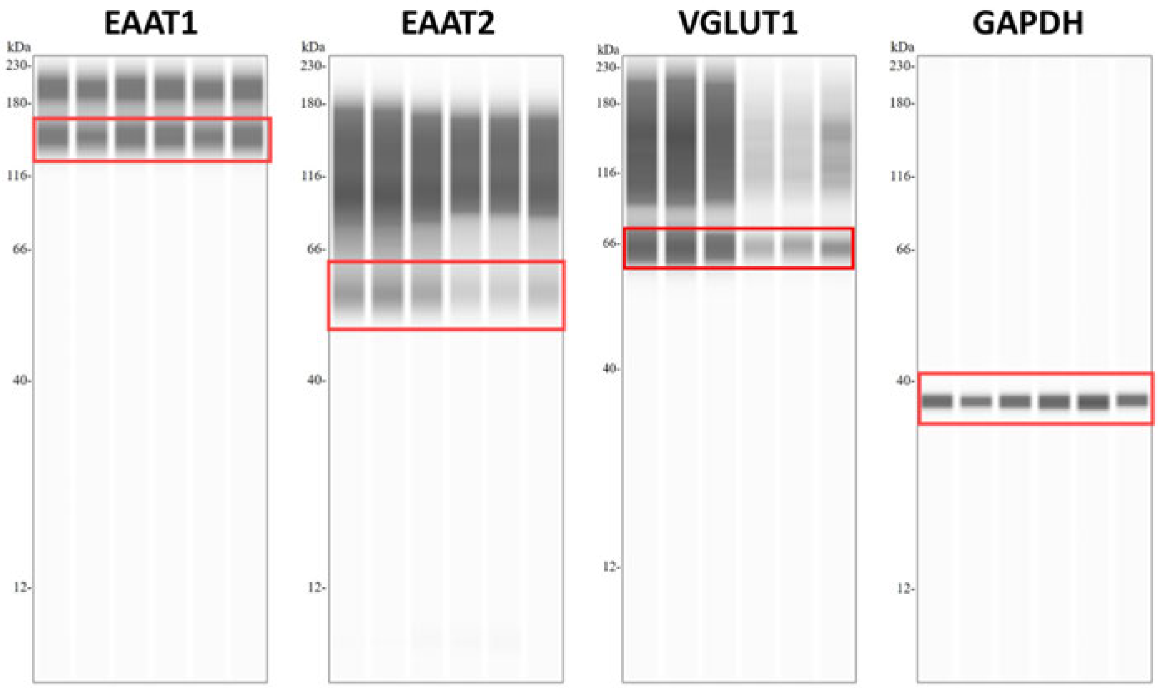

Figure S7. Original Western blot image of Figure 2M.

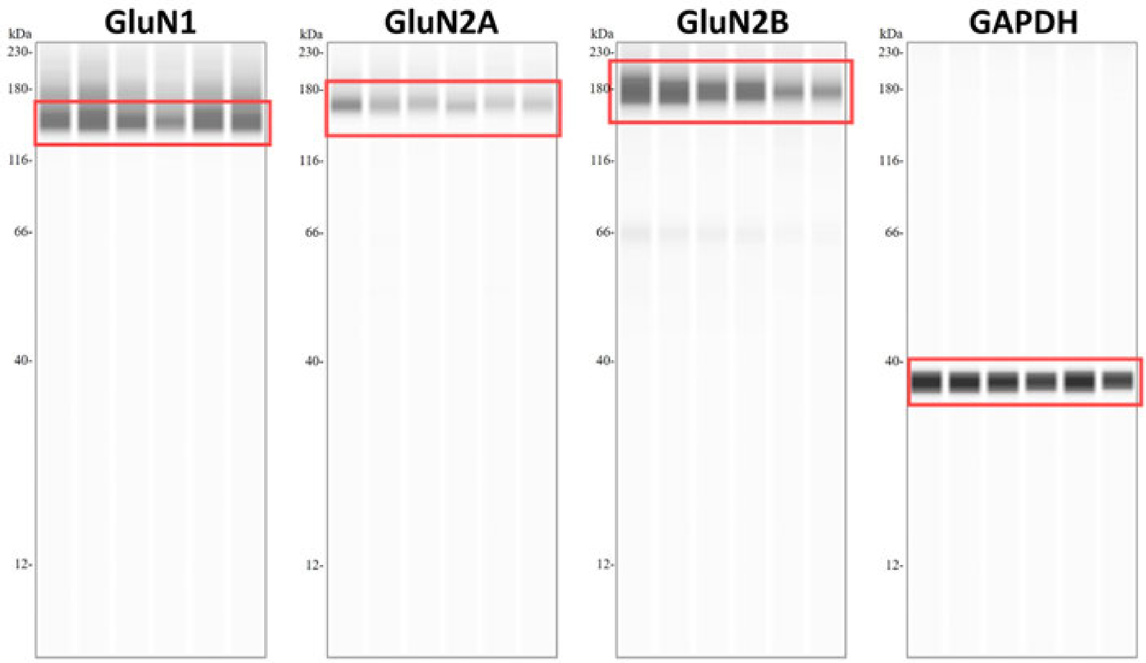

Figure S8. Original Western blot image of Figure 3K.

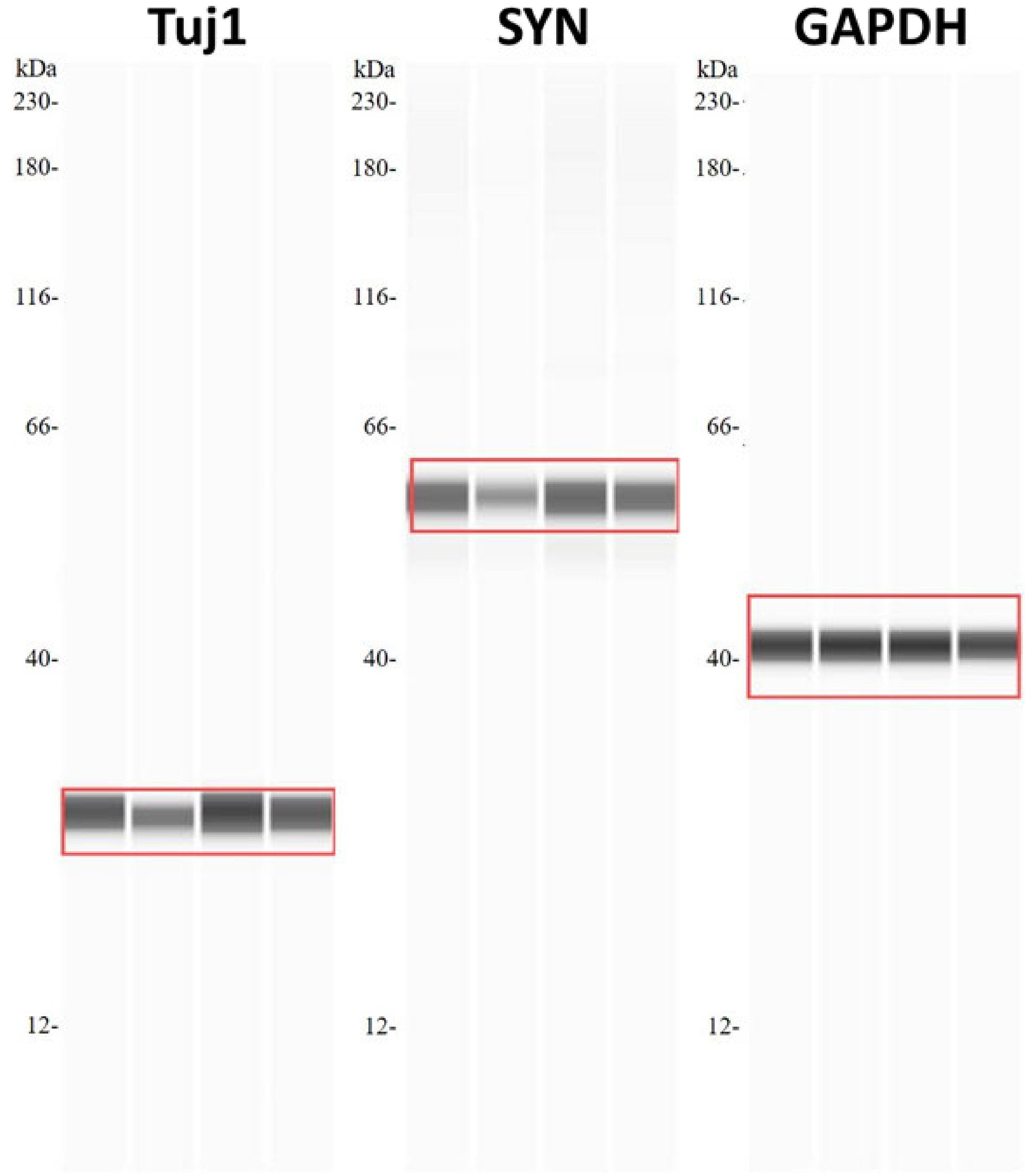

Figure S9. Original Western blot image of Figure 4C.
